# Transmission Model of Hepatitis B Virus with the Migration Effect

**DOI:** 10.1155/2013/150681

**Published:** 2013-06-24

**Authors:** Muhammad Altaf Khan, Saeed Islam, Muhammad Arif, Zahoor ul Haq

**Affiliations:** ^1^Department of Mathematics, Abdul Wali Khan University, Mardan, Khyber Pakhtunkhwa, Pakistan; ^2^Department of Management Sciences, Abdul Wali Khan University, Mardan, Khyber Pakhtunkhwa, Pakistan

## Abstract

Hepatitis B is a globally infectious disease. Mathematical modeling of HBV transmission is an interesting research area. In this paper, we present characteristics of HBV virus transmission in the form of a mathematical model. We analyzed the effect of immigrants in the model to study the effect of immigrants for the host population. We added the following flow parameters: “the transmission between migrated and exposed class” and “the transmission between migrated and acute class.” With these new features, we obtained a compartment model of six differential equations. First, we find the basic threshold quantity Ro and then find the local asymptotic stability of disease-free equilibrium and endemic equilibrium. Furthermore, we find the global stability of the disease-free and endemic equilibria. Previous similar publications have not added the kind of information about the numerical results of the model. In our case, from numerical simulation, a detailed discussion of the parameters and their numerical results is presented. We claim that with these assumptions and by adding the migrated class, the model informs policy for governments, to be aware of the immigrants and subject them to tests about the disease status. Immigrants for short visits and students should be subjected to tests to reduce the number of immigrants with disease.

## 1. Introduction

According to World Health Organization, about 350 million people are infected with the hepatitis B virus (HBV)^1^ and about 170 million people are chronically infected with the hepatitis C virus (HCV)^2^. The majority of those infected live in developing countries with few incidences in Western countries. Migration is one of the defining issues of our time. For example, more than 5 million Canadians migrate out of the country each year and over 250,000 new immigrants arrive in Canada each year. Countries of the world are increasingly connected through travel and migration, and thus, migration has health implications in one location for both local and global migrations, since infectious diseases do not remain isolated geographically. The UK Hepatitis Foundation estimated in 2007 that the number of hepatitis B cases in the UK doubled in the previous 6 years chiefly due to immigration of infected people, many from the new member states of the European Union where the prevalence of viral hepatitis is higher. The United Arab Emirates mandated that hepatitis C testing is to be done at the time of residence visa renewals and added hepatitis C to the list of diseases such as HIV and hepatitis B as diseases warranting deportation [1]. In China, hepatitis B virus infection is a major public health problem. Hepatitis B is the first one among the diseases with legal management measures. In China, an estimated 93 million people have been infected with the hepatitis B virus [2]. The seroepidemiological survey on HBV infection conducted in 2006 showed that HBsAg carrier rate was 7.18 percent in the overall dynamics of HBV. In this paper, we consider a system of ordinary differential equations which describes the transmission of HBV transmission in China. Several mathematical models have been formulated on the HBV transmission in China. Medley and coauthors used a mathematical model and developed the strategies to eliminate the HBV in New Zealand in 2008 [3, 4]. Anderson and May [5] used a mathematical model which illustrated the effects of carriers on the transmission of HBV. An age structure model was proposed by Zhao et al. [6] to predict the dynamics of HBV transmission and evaluate the long-term effectiveness of the vaccination program in China. Wang et al. [7] proposed and analyzed the hepatitis B virus infection in a diffusion model confined to a finite domain. A hepatitis B virus (HBV) model with spatial diffusion and saturation response of the infection rate is investigated by Xu and Ma [8]. Also, Zou et al. [9] proposed a mathematical model to understand the transmission dynamics and prevalence of HBV in mainland China. A model to describe waning of immunity after sometime has been studied by a number of authors [10–16]. In the context of rapid global migration, there is a potential for any disease to be transferred faster than was previously possible. These implications concerning the movement of HBV and HCV merit far more attention by countries and the international community than they have given the problem to date. This is especially important given that the scope and speed of migration is expected to grow in coming years. In the context of rapid global migration, there is a potential for any disease to be transferred faster than was previously possible. These implications concerning the movement of HBV and HCV merit far more attention by countries and the international community than they have given the problem to date. This is especially important given that the scope and speed of migration is expected to grow in coming years.

In this paper, we construct the compartmental model of hepatitis B transmission. We have categorized the model into six compartments: Susceptible-*S*(*t*), Exposed-*E*(*t*), Acute-*A*(*t*), Carrier-*C*(*t*), Vaccinated-*V*(*t*), and Migrated-*M*(*t*) individuals. The migrated class of individuals comes from different parts of the world to the host country, and their interaction occurs in the form of sexual interactions, blood transportation, and transfusion. We modify the model from Pang et al. [10] by adding some new transmission dynamics and introduce the migrated class in the model. Furthermore, some authors [11, 13, 15, 17] show that acute hepatitis B could be found today in newborns of infected mothers. Pang et al. [10] added the vertical transmission term to exposed class from chronic carriers class on the basis of the characteristics of HBV transmission. In this paper, we improved the model of [10], with these new features, by adding the migrated class *M*(*t*) and the following parameters:

- *μ*_1_: the transmission rate from migrated class to exposed class,
- *μ*_2_: the transmission rate from migrated to acute class,
- *δ*: the death rate at the migrated class.

The paper is organized as follows.  Section 2 is devoted to the mathematical formation of the model. In Section 3 we find the Basic Reproduction Number, the disease-free equilibrium, and endemic equilibrium of the proposed model. Local asymptotic stability of the disease-free equilibrium and endemic equilibrium is discussed in Section 4. In Section 5, we study the global asymptotic stability of disease-free and endemic equilibria using the Lyapunov function. In Section 6, we study the numerical results of the proposed model and present the results in the form of plots for illustrations. The conclusion and references are presented in Section 7.

## 2. Model Formulation

**Figure 1:**
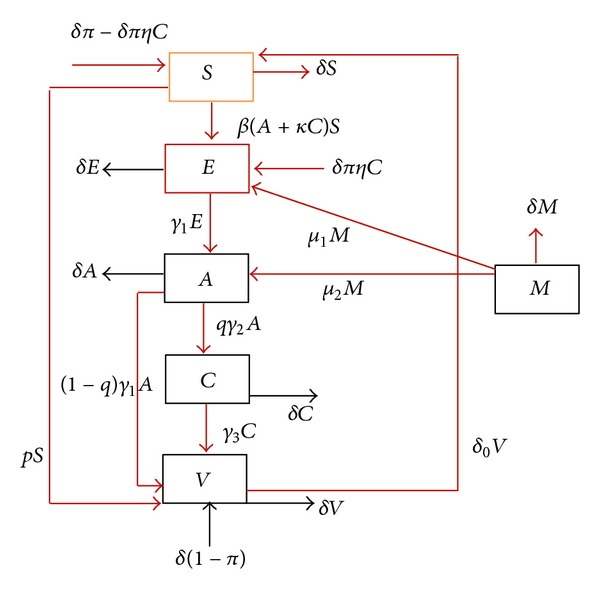
The complete flow diagram of hepatitis B virus transmission model.

 In this section, we present the mathematical formulation of the compartmental model of hepatitis B, which consists of a system of differential equations. The model is based on the characteristics of HBV transmission. We divide the total population into six compartments, that is, Susceptible individuals *S*(*t*), exposed *E*(*t*), Acute *A*(*t*), Carrier *C*(*t*), Immunity class *V*(*t*), and Migrated *M*(*t*). The flow diagram (Figure 1) and the system is given in the following:

(1) dS(t)dt=δπ(1−ηC(t))−δS(t)−β(A(t)+κC(t))S(t)    +δoV(t)−pS(t),dE(t)dt=β(A(t)+κC(t))S(t)−δE(t)+δπηC(t)    −γ1E(t)+μ1M(t),dA(t)dt=γ1E(t)−δA(t)−qγ2A(t)−(1−q)γ1A(t)    +μ2M(t),dC(t)dt=qγ2A(t)−δC(t)−γ3C(t),dV(t)dt=γ3C(t)+(1−q)γ1A(t)−δoV(t)−δV(t)    +δ(1−π)+pS(t),dM(t)dt=−(μ1+μ2)M(t)−δM(t).

Subject to the initial conditions,

(2) S(0)≥0, E(0)≥0, A(0)≥0,C(0)≥0, V(0)≥0, M(0)≥0.

The proportion of failure immunization is shown by *π*. *δ* represent both the death and birth rate. At the *γ*_1_ rate the exposed individuals become infectious and move to the Acute class. *γ*_2_ is the rate at which the individuals move to the carrier class, *γ*_3_ is the flow of carrier to vaccinated class, *β* shows the transmission coefficient, *κ* represents the carrier infectiousness to acute infection, *q* is the proportion of acute individuals that become carrier, *δ*_*o*_ represent the loss of immunity rate and the individual become the susceptible again, *p* represents the vaccination of susceptible individuals, *μ*_1_ represents the rate of flow from migrated class to exposed class, and *γ*_2_ is the rate of transmission from migrated class to acute class. *η* is the unimmunized children born to carrier mothers, *δ*(1 − *π*) measures the successful immunization of newborn babies, and the term *δπ*(1 − *ηC*(*t*)) shows that the newborns are unimmunized and become susceptible again. 

We assume that the total population size is equal to 1, and just for simplifications, *S*(*t*) is the susceptible, *E*(*t*) the exposed, *A*(*t*) the acute, *C*(*t*) the carrier, *V*(*t*) the immunity, and *M*(*t*) is the migrated class representing the state variables in our proposed population model. The sum of the total population is *S*(*t*) + *E*(*t*) + *A*(*t*) + *C*(*t*) + *V*(*t*) + *M*(*t*) = 1, holds. We just add (1), and we can easily get. We ignore the fifth equation in system (1); so, the new models become

(3) dS(t)dt=δπ(1−ηC(t))−δS(t)−β(A(t)+κC(t))S(t)+δo(1−(S(t)+E(t)+A(t)+C(t)+M(t)))−pS(t),dE(t)dt=β(A(t)+κC(t))S(t)−δE(t)+δπηC(t)    −γ1E(t)+μ1M(t),dA(t)dt=γ1E(t)−δA(t)−qγ2A(t)−(1−q)γ1A(t)    +μ2M(t),dC(t)dt=qγ2A(t)−δC(t)−γ3C(t),dM(t)dt=−(μ1+μ2)M(t)−δM(t).

Let

(4) Γ={(S,E,A,C,M)∈ℜ+5, ∣ S(t)≤δπ+δoδ+δo+p,   (S+E+A+C+M≤δπ+δoδ+δo)}.

Here, Γ is a positively invariant set. All the solutions lie inside Γ which is our main focus. 

## 3. Basic Reproduction Number/Threshold Quantity

 The basic reproduction number or the threshold quantity *ℜ*_0_ for the proposed model gives an average number of secondary infection when an infection is introduced in a purely susceptible population. We use the idea developed by [18], and also for detail see [19]. We have

(5) $$\begin{array}{c} ℱ=\left[\begin{array}{ccc} 0 & \beta S^{o} & \beta \kappa S^{o}\\ 0 & 0 & 0\\ 0 & 0 & 0 \end{array}\right],\;\;V=\left[\begin{array}{ccc} \delta +\gamma_{1} & 0 & -\delta \pi \eta \\ -\gamma_{1} & \delta +q\gamma_{2}+\left(1-q\right)\gamma_{1} & 0\\ 0 & -q\gamma_{2} & \delta +\gamma_{3} \end{array}\right],\\ V^{-1}=\frac{\left[\begin{array}{ccc} \left(\delta +q\gamma_{2}+\left(1-q\right)\gamma_{1}\right)\left(\delta +\gamma_{3}\right) & q\gamma_{2}\delta \pi \eta & \delta \pi \eta \left(\delta +q\gamma_{2}+\left(1-q\right)\gamma_{1}\right)\\ \gamma_{1}\left(\delta +\gamma_{3}\right) & \left(\delta +\gamma_{1}\right)\left(\delta +\gamma_{3}\right) & \delta \pi \eta \gamma_{1}\\ q\gamma_{1}\gamma_{2} & q\gamma_{2}\left(\delta +\gamma_{1}\right) & \left(\delta +\gamma_{1}\right)\left(\delta +q\gamma_{2}+\left(1-q\right)\gamma_{1}\right) \end{array}\right]}{\left(\delta +\gamma_{1}\right)\left(\delta +q\gamma_{2}+\left(1-q\right)\gamma_{1}\right)\left(\delta +\gamma_{3}\right)-\delta \pi \eta q\gamma_{1}\gamma_{2}}, \end{array}$$

where *Q*_1_ = (*δ* + *qγ*_2_ + (1 − *q*)*γ*_1_) and
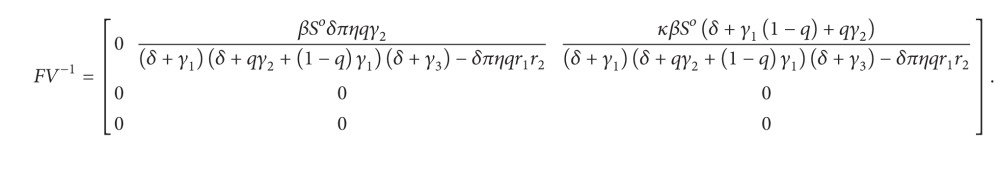
(6)
So, the reproduction number given by *ρ*(*FV* ^−1^) is

(7) $$\begin{array}{c} {ℜ}_{o}=\frac{\kappa \beta S^{o}\left(\delta +\gamma_{1}\left(1-q\right)+q\gamma_{2}\right)+\delta \pi \eta qr_{1}r_{2}}{\left(\delta +\gamma_{1}\right)\left(\delta +q\gamma_{2}+\left(1-q\right)\gamma_{1}\right)\left(\delta +\gamma_{3}\right)}. \end{array}$$

Here, *S* ^*o*^ shows the disease-free equilibrium (DFE), and *D*_*o*_ = (*S* ^*o*^, 0,0, 0,0), giving *S* ^*o*^ = (*δπ* + *δ*_*o*_)/(*δ* + *δ*_*o*_ + *p*). The endemic equilibrium point T* = (S*, E*, A*, C*, M*) for system (3), whose endemic equilibrium is given in the following subsection.

*Endemic Equilibria.* To find the endemic equilibria of the system (3), by setting *S* = S*, *E* = E*, *A* = A*, *C* = C*, and *M* = M*, equating left side of the system (3) equal to zero, we obtained

(8) S∗=Q1(δ+γ1)(δ+γ3)(1−R0)+κβSoQ1γ1β((δ+γ3)+κqγ2)  −(δ+γ3)[(δ+γ1)+μ1γ1]M∗γ1β((δ+γ3)+κqγ2)A∗,E∗=Q1A∗−μ2M∗γ1,C∗=qγ2(δ+γ3)A∗.

## 4. Local Stability Analysis

 In this section, we find the local stability of disease-free and endemic equilibria. First, we show the local asymptotical stability of DFE equilibrium, and then we find the local asymptotical stability of endemic equilibrium. Now, we show the local stability of DFE about the point *D*_*o*_ = (*S* ^*o*^, 0,0, 0,0) in the following theorem.

**Theorem 1**

For *R*_0_ ≤ 1, the disease-free equilibrium of the system (3) about an equilibrium point *D*_*o*_ = (*S* ^*o*^, 0,0, 0,0) is locally asymptotically stable if ((*Q*_1_(*δ* + *δ*_*o*_ + *p*)(*δ* + *γ*_1_) > *βγ*_1_(*δπ* + *δ*_*o*_)); otherwise, the disease-free equilibrium of the system (3) is unstable for *R*_0_ > 1.

**Proof**

To show the local stability of the system (3), about the point *D*_*o*_, we set the left-hand side of the system (3) equating to zero, and we obtain the following Jacobian matrix *J*_*o*_(*ζ*):

(9) $$\begin{array}{c} J_{o}\left(\zeta \right)=\left(\begin{array}{ccccc} -\delta -\delta_{o}-p & \delta_{o} & -\beta S^{o}-\delta_{o} & -\delta \pi \eta -\beta \kappa S^{o}-\delta_{o} & 0\\ 0 & -\left(\delta +\gamma_{1}\right) & \beta S^{o} & \beta \kappa S^{o}+\delta \pi \eta & \mu_{1}\\ 0 & \gamma_{1} & -Q_{1} & 0 & \mu_{2}\\ 0 & 0 & q\gamma_{2} & -\left(\delta +\gamma_{3}\right) & 0\\ 0 & 0 & 0 & 0 & -\left(\mu_{1}+\mu_{2}+\delta \right) \end{array}\right). \end{array}$$

By the elementary row operation, we get the following matrix:

(10) $$\begin{array}{c} J_{o}\left(\zeta \right)=\left(\begin{array}{ccccc} -\left(\delta +\delta_{o}+p\right) & -\delta_{o} & -\beta S^{o}-\delta_{o} & -\delta \pi \eta -\beta \kappa S^{o} & 0\\ 0 & -\left(\delta +\gamma_{1}\right) & \beta S^{o} & \beta \kappa S^{o}+\delta \pi \eta & \mu_{1}\\ 0 & 0 & -Q_{1}\left(\delta +\gamma_{1}\right)+\gamma_{1}\beta S^{o} & \gamma_{1}\left(\beta \kappa S^{o}+\delta \pi \eta \right) & \left(\delta +\gamma_{1}\right)\mu_{2}+\mu_{1}\gamma_{1}\\ 0 & 0 & 0 & T_{1} & 0\\ 0 & 0 & 0 & 0 & -\left(\mu_{1}+\mu_{2}+\delta \right) \end{array}\right), \end{array}$$

where *Q*_1_ = (*δ* + *qγ*_2_ + (1 − *q*)*γ*_1_) and *T*_1_ = −(−*Q*_1_(*δ* + *γ*_1_) + *γ*_1_ *βS* ^*o*^) − *qγ*_1_ *γ*_2_(*βκS* ^*o*^ + *δπ* *η*). The characteristic equation to the previous Jacobian matrix is given by

(11) $$\begin{array}{c} \left(\lambda +\delta +\delta_{o}+p\right)\left(\lambda +\mu_{1}+\mu_{2}+\delta \right)\left(\lambda^{3}+a_{1}\lambda^{2}+a_{2}\lambda +a_{3}\right)=0. \end{array}$$

The first two eigenvalues −(*δ* + *δ*_*o*_ + *p*) and −(*μ*_1_ + *μ*_2_ + *δ*) have negative real parts. For the rest of the eigenvalues, we get

(12) a1=(δ+δo+p)(Q1(δ+γ3)+1+Q1)(1−R0)   +β(δπ+δo)((γ1(δ+γ3)+qγ1γ2)−(Q1κ+γ1))   ×(δ+δo+p)−1,a2=(Q1(δ+γ1)(δ+δo+p)−βγ1(δπ+δo))  ×{(δ+δo+p)[(δ+γ1)+(δ+γ1)2]   +(δ+γ1)[Q1(δ+γ1)(δ+δo+p)−βγ1(δπ+δo)]   + qγ1γ2(δ+γ1)[βκ(δπ+δo)+δπη(δ+δ+p)]}  ×(δ+δo+p)−2  +{qγ1γ2(δ+γ1)(βκ(δπ+δo)+δπη(δ+δo+p))}  ×(δ+δo+p)−1,a3=(δ+γ1)(δ+δo+p)(Q1(δ+δo+p)(δ+γ1)−βγ1(δπ+δo))   ×[qγ1γ2(βκ(δπ+δo)+δπη)    + (Q1(δ+γ1)(δ+δo+p)−βγ1(δπ+δo))].

By the Routh-Hurwitz criteria, *a*_1_ > 0, *a*_3_ > 0, and *a*_1_ *a*_2_ > *a*_3_. Here, *a*_1_ > 0 when *R*_0_ ≤ 1 and ((*Q*_1_(*δ* + *δ*_*o*_ + *p*)(*δ* + *γ*_1_) > *βγ*_1_(*δπ* + *δ*_*o*_)). Also, *a*_2_ > 0 and *a*_3_ > 0, and then *a*_1_ *a*_2_ > *a*_3_. So, according to the Routh-Hurwitz criteria, the Jacobian matrix has negative real parts if and only if *R*_0_ ≤ 1. Thus by Routh-Hurwitz criteria, the DFE of the system (3) is locally asymptotically stable about the point *D*_*o*_ = (*S* ^*o*^, 0,0, 0,0). The proof is completed.

The stability of the disease-free equilibrium for *R*_0_ ≤ 1 means that the disease dies out from the population. Next we show that the endemic equilibrium of the system (3) is locally asymptotically stable for *R*_0_ > 1. When the disease-free equilibrium is locally asymptotically stable for *R*_0_ ≤ 1, then the endemic equilibrium does not exist, but we are interested to know about the properties of an endemic equilibrium for *R*_0_ > 1. 

### 4.1. Stability of Endemic Equilibrium (EE)

 In this subsection, we find the local asymptotic stability of EE about D* = (S*, E*, A*, C*, M*), and we prove the local stability of endemic equilibrium in the following.

**Theorem 2**

For *R*_0_ > 1, the endemic equilibrium D* of system (3) is locally asymptotical stable, if the following conditions hold:

(13) $$\begin{array}{c} \gamma_{1}Z_{2}\delta_{o}{S^{*}}^{2}>\gamma_{1}\beta^{2}\kappa ,\\ G_{1}>G_{2}; \end{array}$$

otherwise, the system is unstable.

**Proof**

Here, we prove the that the system (3) about the equilibrium point D* is locally stable, and for this, we obtain the Jacobian matrix J*(*ζ*) of the system (3), in the following:

(14) $$\begin{array}{c} J^{*}\left(\zeta \right)=\left(\begin{array}{ccccc} -\left(\delta +\delta_{o}+p+\beta \left(A^{*}+\kappa C^{*}\right)\right) & \delta_{o} & -\beta S^{*}-\delta_{o} & -\delta \pi \eta -\beta \kappa S^{*}-\delta_{o} & -\delta_{o}\\ \beta \left(A^{*}+\kappa C^{*}\right) & -\left(\delta +\gamma_{1}\right) & \beta S^{*} & \beta \kappa S^{*}+\delta \pi \eta & \mu_{1}\\ 0 & \gamma_{1} & -Q_{1} & 0 & \mu_{2}\\ 0 & 0 & q\gamma_{2} & -\left(\delta +\gamma_{3}\right) & 0\\ 0 & 0 & 0 & 0 & -\left(\delta +\mu_{1}+\mu_{2}\right) \end{array}\right). \end{array}$$

By elementary row operation and after simplification, we get the following Jacobian matrix:

(15) $$\begin{array}{c} J^{*}\left(\zeta \right)=\left(\begin{array}{ccccc} -Z_{1} & -\delta_{o} & -\beta S^{*}-\delta_{o} & Z_{3} & -\delta_{o}\\ 0 & -Z_{1}\left(\delta +\gamma_{1}\right)-Z_{2}\delta_{o} & Z_{1}\beta S^{*}-Z_{2}\left(\beta S^{*}+\delta_{o}\right) & Z_{1}Z_{4}+Z_{2}Z_{3} & Z_{1}\mu_{1}-Z_{2}\delta_{o}\\ 0 & 0 & Z_{5} & \gamma_{1}\left(Z_{1}Z_{4}+Z_{2}Z_{3}\right) & Z_{6}\\ 0 & 0 & 0 & Z_{7} & 0\\ 0 & 0 & 0 & 0 & -\left(\mu_{1}+\mu_{2}+\delta \right) \end{array}\right), \end{array}$$

where

(16) Z1=δ+δo+p+β(A∗+κC∗),Z2=β(A∗+κC∗),Z3=−(δπη+βκS∗+δo),Z4=(βκS∗+δπη),Z5=− Q1(−Z1(δ+γ1)−Z2δo)   +γ1(Z4βS∗−Z2(βS∗+δo)),Z6=−μ2(−Z1(δ+γ1)−Z2δo)+γ1(Z1μ1−Z2δo),Z7=−Z5(δ+γ3)−Z5γ1(Z1Z4+Z2Z3).

The eigenvalue *λ*_1_ = −(*μ*_1_ + *μ*_2_ + *δ*) < 0, *λ*_2_ = −*Z*_1_, and using the value of *Z*_1_ and further C*, we get *λ*_2_ = −(*δ* + *δ*_*o*_ + *p* + *β*((*δ* + *γ*_3_) + *κ* *qγ*_2_)A*) < 0. *λ*_3_ = −(*Z*_1_(*δ* + *γ*_1_) + *Z*_2_ *δ*_*o*_) < 0, as *Z*_1_ > 0 and *Z*_2_ > 0. *λ*_4_ = *Z*_5_, *λ*_4_ < 0, if and only if *Z*_5_ < 0. After the simplifications, we get

(17) β∗A∗(γ1Z2δo−γ1β2κS∗2) +{γ1βδπηβ∗∗∗M∗  −[(γ1βδπηβ∗∗+β∗Q1)Z1(δ+γ1)   +Q1Z2δoβ∗]}>0,

where β* = *γ*_1_ *β*((*δ* + *γ*_3_) + *κ* *qγ*_2_), β** = *Q*_1_(*δ* + *γ*_1_)(*δ* + *γ*_3_)(1 − *R*_0_) + *κ* *βS* ^*o*^ *Q*_1_, and {*γ*_1_ *β* *δπ* *η* β***M* > [(*γ*_1_ *β* *δπ* *η* β** + β*Q_1_)*Z*_1_(*δ* + *γ*_1_) + *Q*_1_ *Z*_2_ *δ*_*o*_ β*]}, say *G*_1_ = *γ*_1_ *β* *δπ* *η* β***M* and *G*_2_ = [(*γ*_1_ *β* *δπ* *η* β** + β*Q_1_)*Z*_1_(*δ* + *γ*_1_) + *Q*_1_ *Z*_2_ *δ*_*o*_ β*]β*** = (*δ* + *γ*_3_)[(*δ* + *γ*_1_) + *γ*_1_ *μ*_1_]. So, *λ*_4_ has negative real part if (*γ*_1_ *Z*_2_ *δ*_*o*_ > *γ*_1_ *β* ^2^ κS*^2^). For *λ*_5_ = *Z*_7_, we obtained negative real parts, and by using the *Z*_5_ which is positive under the conditions described in *λ*_4_, and *Z*_1_ > 0, *Z*_2_ > 0, we get the negative real parts. Thus, all the eigenvalues have negative real parts, so by the Routh-Hurwitz criteria the endemic equilibrium point D* is locally asymptotically stable when *R*_0_ > 1. The proof is completed.

## 5. Global Stability of DFE

 In this section, we present the global stability of disease-free equilibrium DFE of the system (3). For different biological model, the Lyapunov function was used by [20, 21] for the global stability. For our model, we define and construct Lyapunov function in the following for the global stability of DFE. Further the global stability of endemic equilibrium we use the Lyapunov function and find its global asymptotical stability.

**Theorem 3**

For *R*_0_ ≤ 1, the disease-free equilibrium of the system (3) is stable globally asymptotically, if *S* = *S* ^*o*^ and unstable for *R*_0_ > 1.

**Proof**

Here, we define the Lyapunov function for the global stability of disease-free equilibrium, given by

(18) $$\begin{array}{c} V\left(t\right)=\left[d_{1}\left(S-S^{o}\right)+d_{2}E+d_{3}A+d_{4}C+d_{5}M\right]. \end{array}$$

Differentiating the previous function with respect to *t* and using the system (3),

(19) V′(t)=d1S′+d2E′+d3A′+d4C′+d5M′,V′(t)=d1[δπ(1−ηC(t))−δS(t)−β(A(t)+κC(t))S(t)   + δo(1−(S(t)+E(t)+A(t)+C(t)+M(t)))−pS(t)] +d2[β(A(t)+κC(t))S(t)−δE(t)+δπηC(t)    − γ1E(t)+μ1M(t)] +d3[γ1E(t)−δA(t)−qγ2A(t)−(1−q)γ1A(t)   + μ2M(t)] +d4[qγ2A(t)−δC(t)−γ3C(t)] +d5[−(μ1+μ2)M(t)−δM(t)],

where *d*_*i*_, *i* = 1,2,…5, are some positive constants to be chosen later.

After the arrangement, we obtain

(20) V′(t)=[d2−d1](β(A+κC))S+[d2−d1]δπηC    +[d3γ1−d2(δ+γ1)]E+[d2μ1−d5δ]M    +[d4qγ2−d3Q1]A+d1δπ−d1δS+d1δo    −d1δo(S+E+A+C+M)−d1pS    −d4(δ+γ3)C.

Choosing the constants, *d*_1_ = *d*_2_ = *γ*_1_,  *d*_3_ = (*δ* + *γ*_1_),  *d*_4_ = *Q*_1_(*δ* + *γ*_1_)/*qγ*_2_, and *d*_5_ = *γ*_1_ *μ*_1_/*δ*.

After the simplification, we get

(21) V′(t)=−(S−So)−γ1δoE−γ1δoA    −(γ1δo+(δ+γ3)(δ+γ1)Q1qγ2)C    −(γ1δo+(δo+γ1)μ2)M,

where *S* ^*o*^ = (*δπ* + *δ*_*o*_)/(*δ* + *δ*_*o*_ + *p*). *V*′(*t*) = 0 if and only if *S* = *S* ^*o*^ and *E* = *A* = *C* = *M* = 0. Also, *V*′(*t*) is negative for (*S* > *S* ^*o*^). So, by [22], the DFE is globally asymptotically stable in Γ. The proof is completed.

### 5.1. Global Stability of Endemic Equilibrium

 In this subsection, we show the global asymptotical stability of the system (3). To do this, we state and prove the following theorem for the global stability of endemic equilibrium.

**Theorem 4**

For *R*_0_ > 1, system (3) is globally asymptotically stable, if *S* = S* and *δ*_*o*_ > *μ*_1_, and unstable for *R*_0_ ≤ 1.

**Proof**

To prove that system (3) is globally asymptotically stable, we define the Lyapunov in the following:

(22) L(t)=(μ1+μ2+μ3)γ1γ1+δ(S−S∗)+(μ1+μ2+μ3)γ1γ1+δE    +(μ1+μ2+μ3)A+(μ1+μ2+μ3)Q1qγ2C+μ2M.

Taking the derivative with respect to time *t*, using the system (3),

(23) L′(t)=(μ1+μ2+μ3)γ1γ1+δ    ×[δπ(1−ηC(t))−δS(t)−β(A(t)+κC(t))S(t)     +δo(1−(S(t)+E(t)+A(t)+C(t)+M(t)))     −pS(t)]   +(μ1+μ2+μ3)γ1γ1+δ[β(A(t)+κC(t))S(t)−δE(t)           + δπηC(t)−γ1E(t)+μ1M(t)]   +(μ1+μ2+μ3)[γ1E(t)−δA(t)−qγ2A(t)          −(1−q)γ1A(t)+μ2M(t)]   +(μ1+μ2+μ3)Q1qγ2[qγ2A(t)−δC(t)−γ3C(t)]   +μ2[−(μ1+μ2+δ)M].

Simplifying, we obtained

(24) L′(t)=−(μ1+μ2+μ3)γ1(γ1+δ)(δ+δo+p)(S−S∗)   −(μ1+μ2+μ3)γ1δo(γ1+δ)[E+A+C]   −(μ1+μ2+μ3)γ1(γ1+δ)[δo−μ1]M.

The endemic equilibrium of the system (3) is globally asymptotically stable for *R*_0_ > 1, if *S* = S* and *δ*_*o*_ > *μ*_1_. So, the endemic equilibrium of the system (3) is globally asymptotically stable. The proof is completed.

## 6. Numerical Simulations

**Figure 2:**
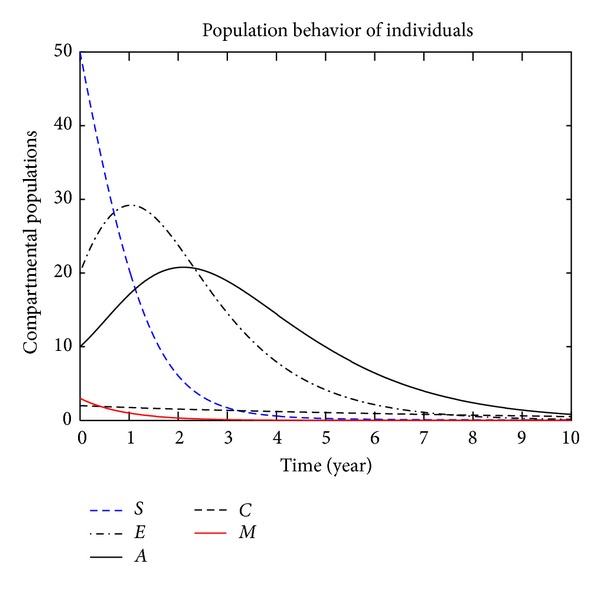
The plot shows the HBV transmission model of hepatitis B, with *μ*_1_ = 0.90 and *μ*_2_ = 0.90.

**Figure 3:**
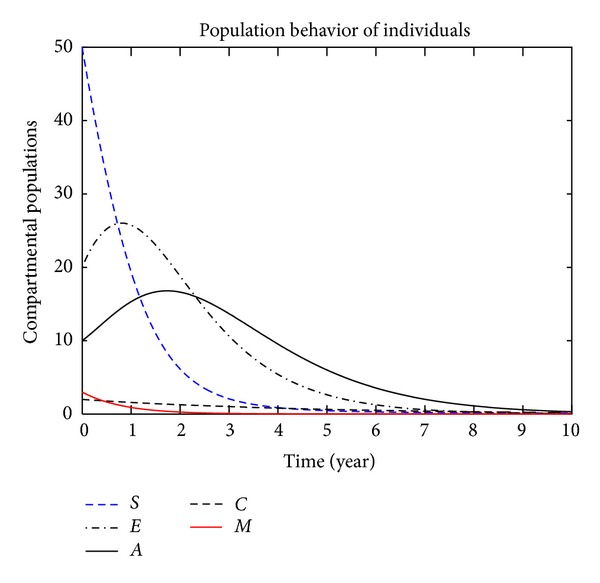
The plot shows the HBV transmission model of hepatitis B, with *μ*_1_ = 0.80 and *μ*_2_ = 0.90.

**Figure 4:**
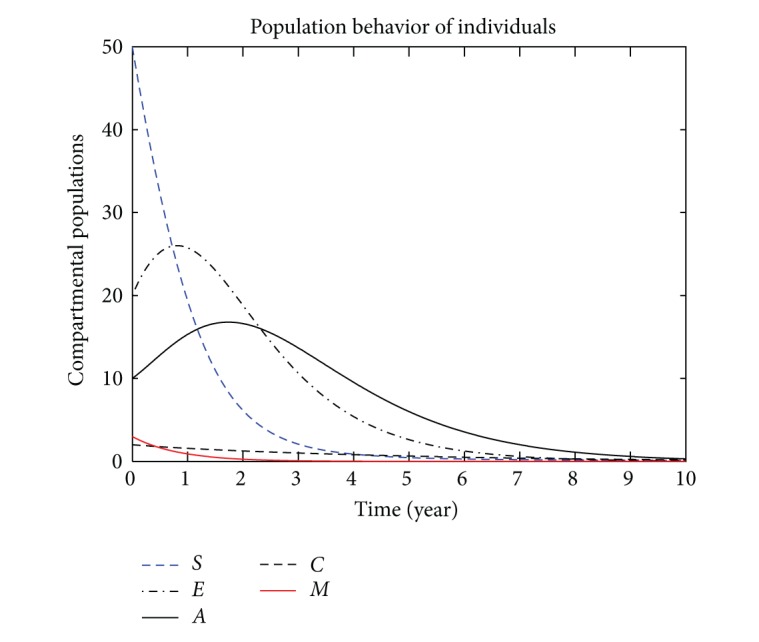
The plot shows the HBV transmission model of hepatitis B, with *μ*_1_ = 0.70 and *μ*_2_ = 0.80.

**Figure 5:**
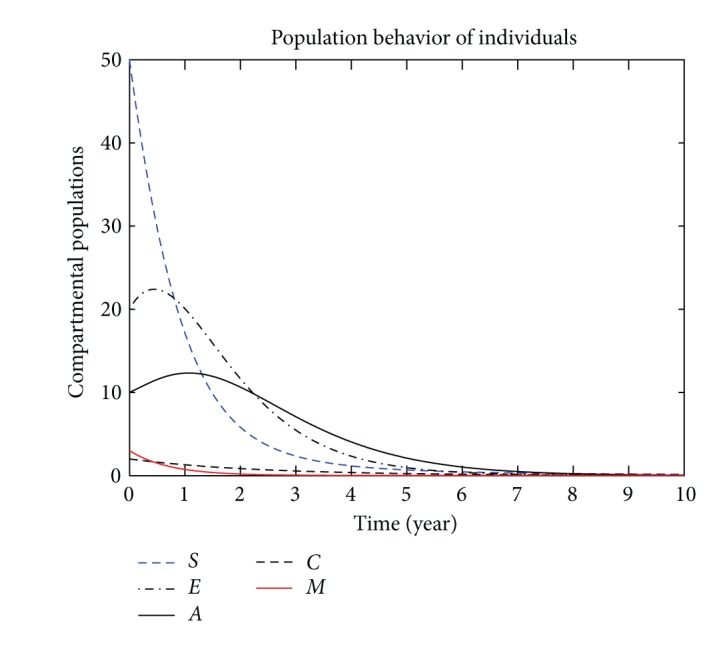
The plot shows the HBV transmission model of hepatitis B, with *μ*_1_ = 0.50 and *μ*_2_ = 0.60.

**Figure 6:**
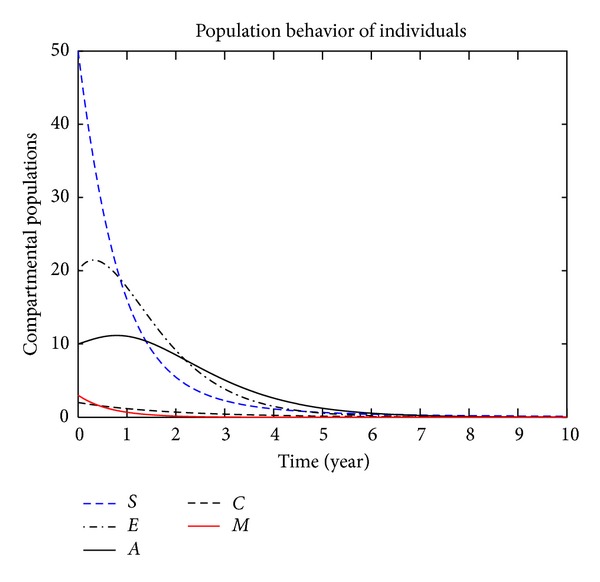
The plot shows the HBV transmission model of hepatitis B, with *μ*_1_ = 0.20 and *μ*_2_ = 0.30.

**Figure 7:**
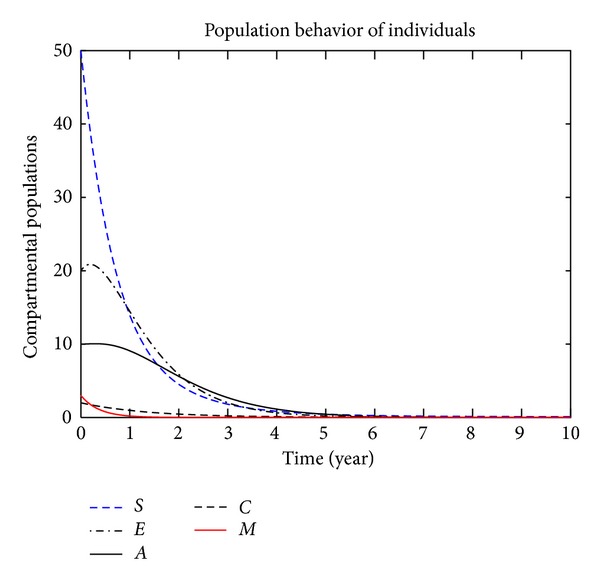
The plot shows the HBV transmission model of hepatitis B, with *μ*_1_ = 0.20 and *μ*_2_ = 0.40.

**Figure 8:**
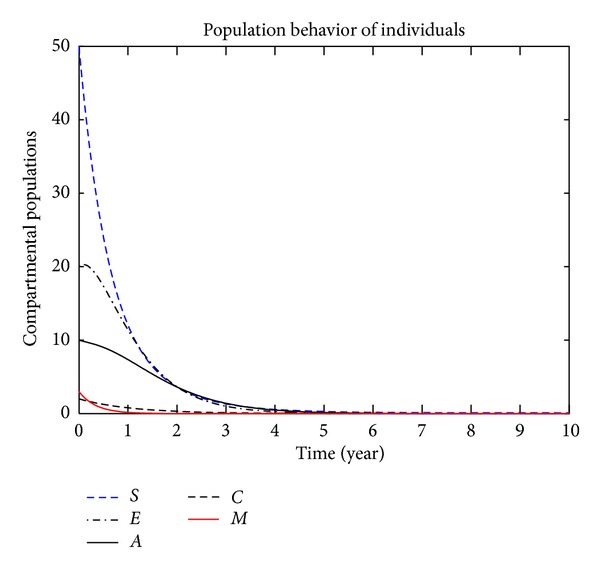
The plot shows the HBV transmission model of hepatitis B, with *μ*_1_ = 0.10 and *μ*_2_ = 0.20.

 In this section, we present the numerical simulation of the proposed model (3), by using the Runge-Kutta order four scheme. For different values of the parameters, the numerical results are presented in Figures 2, 3, 4, 5, 6, 7, and 8. The variation of migration parameters *μ*_1_ and *μ*_2_, with different values, is presented. In the numerical solution of the model, the parameters and their values are presented in Table 1. In our simulation, the susceptible individuals are shown by dashed line, the exposed individuals by dotted dashed, the acute individuals by bold line, the carrier by dashed line and the migrated individuals by red bold line. Figures 2 to 8 represent the compartmental population of hepatitis B individuals with migration effect. The values presented in Table 1 are fixed except for *μ*_1_ and *μ*_2_. In Figure 2, by the values for *μ*_1_ = 0.9 and *μ*_2_ = 0.9, we see that the population of exposed and acute individuals is increasing. In Figure 3, we set *μ*_1_ = 0.8 and *μ*_2_ = 0.9, and the population of exposed and acute individuals decreased. Decreasing the values of *μ*_1_ and *μ*_2_, we obtain different results; see Figures 2 to 8. When we decrease the population of immigrants who have the HBV virus, we see the decrease in the population of exposed and acute individuals. The parameters presented in Table 1 were used by different authors, for example, the natural death rate equally birth rate by [4], the rate at which the latent individuals become infectious by [3], the rate at which the individuals move to the carrier class by [3], the rate at which the individuals move to the carrier class [3], the transmission coefficient *β* by [24], *δ*_*o*_ the loss of immunity rate by [13], the value of *η* by [13], the value of *κ* by [10], and *π* and *q* from [23]. We assume the values for the parameters *γ*_3_, *p*, *μ*_1_, and *μ*_2_ in our simulation.

## 7. Conclusion

 A compartmental model of HBV transmission virus has been presented. A mathematical model has been obtained by adding (1) the migrated class, (2) HBV transmission rate between migrated class and exposed class, (3) transmission rate between migrated class and acute class, and (4) death rate of individuals in the migrated class. By adding these new features, we have obtained a compartmental model of HBV with migration effect. First, we obtained the basic reproduction number for the proposed model. The disease-free equilibrium is locally as well as globally asymptotically stable for *R*_0_ ≤ 1. We obtained the local and global asymptotical stability for the endemic equilibrium. For the reproduction number *R*_0_ > 1, the endemic equilibrium is, locally as well as globally, asymptotically stable. Furthermore, we have solved the compartment model numerically, and the results are presented in Figures 2 to 8. By changing the values of *μ*_1_ and *μ*_2_, different results have been obtained. It is concluded that when the value of *μ*_1_ and *μ*_2_ decreases, the population of (exposed, acute, and carrier) individuals also decrease. The proportion of infected individuals decreases when the proportion of migrated individuals (who have the HBV virus) decreases. So, the number of infected individuals is directly proportional to the number of migrated individuals.

Last yet not the least, the authors of this work have agreed to devise in a course of time a more advanced model on restraining HBV transmission through migration. 

## Figures and Tables

**Table 1:** Parameter values used in numerical simulations.

Notation	Parameter description	Range	Source
*δ*	Natural death rate equally birth rate	0.0143	[4]
*π*	The failure immunization	0-1	[23]
*γ*_1_	The rate at which the latent individuals become infectious	6	[3]
*γ*_2_	The rate at which the individuals move to the carrier class	4	[3]
*γ*_3_	The rate of flow from carrier to the vaccinated class	0.34	Assumed
*β*	The transmission coefficient	0.8	[24]
*q*	The proportion of individuals become carrier	0.005	[23]
*δ*_*o*_	Represent the loss of immunity	0.06–0.03	[13]
*p*	Represent the vaccination of susceptible	0.3	Assumed
*μ*_1_	The rate of flow from Migrated class to exposed class	0.23	Assumed
*μ*_2_	The rate of flow from Migrated class to acute class	0.56	Assumed
*η*	Unimmunized children born to carrier mothers	0.7	[13]
*κ*	The infectiousness of carriers related to acute infection	0-1	[10]
